# Improving the Completeness of Surgical Consent Documentation Through a Closed-Loop Quality Improvement Audit at a Secondary Care Teaching Hospital

**DOI:** 10.7759/cureus.103415

**Published:** 2026-02-11

**Authors:** Hashim Homaida, Zubaida kamal Abdalkhaliq Ahmed, Ahmed Abdelal, Shahd A Awad, Gesaim Mohammed, Hisham Osman Adam Suliman, Elshayma Mohamed Abdalla Edris, Adam Salama Mohamed Adam, Mustafa Mohamed, Musaab Ahmed Ali Fadul, Rouba Hamza Ali, Asma Ahmed, Matab A Abdalla, Mohey Aldien Ahmed Elamin Elnour, Inas Hafiz Ibrahim, Tasneem M Yousif, Abubakr Muhammed, Mohamed Adel Abdalla Mohamed

**Affiliations:** 1 Urology, Hamad Medical Corporation, Doha, QAT; 2 Surgery, Almanagil Teaching Hospital, Managil, SDN; 3 General Surgery, Farwaniya Hospital, Kuwait, KWT; 4 General Surgery, Almanagil Teaching Hospital, Managil, SDN; 5 Emergency Medicine, Almanagil Teaching Hospital, Managil, SDN; 6 Internal Medicine, Al Neelain University, Khartoum, SDN; 7 General Surgery, Dongola Specialized Hospital, Dongola, SDN; 8 Medicine, Hamad Medical Corporation, Doha, QAT; 9 Trauma and Orthopaedics, Royal Derby Hospital, Derby, GBR; 10 General and Colorectal Surgery, South Tipperary General Hospital, Clonmel, IRL; 11 Radiology, University of Cape Town, Cape Town, ZAF; 12 Surgery, Sudan Medical Specialization Board, Khartoum, SDN

**Keywords:** clinical audit, documentation, informed consent, patient safety, quality improvement, surgical consent

## Abstract

Background

Incomplete surgical consent documentation undermines informed decision-making, patient safety, and medico-legal protection. Despite clear ethical and professional standards, deficiencies in consent documentation remain common, particularly in resource-limited settings.

Objective

To assess the completeness of surgical consent documentation, implement targeted quality improvement interventions, and evaluate their impact through a closed-loop clinical audit.

Methods

A closed-loop quality improvement audit was conducted at Almanagil Teaching Hospital, Sudan. Two audit cycles were performed, each reviewing 44 consecutive surgical consent forms. The first cycle (July-August 2025) established baseline compliance with predefined consent documentation standards. A structured intervention, including staff education, feedback, and introduction of a revised consent form, was implemented over two months. The second cycle (October-December 2025) reassessed documentation completeness using the same criteria. Data were analysed descriptively and reported as frequencies and percentages.

Results

At baseline, documentation completeness was low in several key domains, including hospital/file number (1.8%), surgical indication (5.4%), explanation of procedure (16.1%), and anaesthetic and surgical risk explanation (1.8% each). Following the intervention, substantial improvements were observed across nearly all components. Documentation of consent, anaesthesia type, physician and witness details, and patient satisfaction confirmation reached 100% compliance. Explanation of anaesthetic and surgical risks increased to 97.7% and 93.2%, respectively. Documentation of possible additional procedures and patient incapacity details improved to 47.7% and 50.0%, although these remained below full compliance.

Conclusion

This audit demonstrates that structured, audit-driven quality improvement interventions can markedly enhance the completeness of surgical consent documentation. Regular auditing and sustained reinforcement are recommended to maintain improvements and address remaining gaps.

## Introduction

A clinical audit is a systematic quality improvement methodology that assesses clinical practice against predefined standards, identifies gaps in performance, and implements targeted changes designed to improve healthcare delivery. Completed audit cycles with re-measurement after intervention are essential to demonstrate whether proposed changes have led to sustained improvement. These are central components of clinical governance and patient safety frameworks in surgical care settings [1].

Informed consent is a fundamental ethical and legal requirement in modern clinical practice, particularly for invasive procedures such as surgery. A valid consent process ensures that patients are adequately informed about the nature, risks, benefits, and alternatives of proposed surgical interventions, supports shared decision-making, and upholds patient autonomy. Consent documentation also serves to protect both patients and healthcare professionals by clearly recording that appropriate communication occurred before a procedure was undertaken [2].

However, studies have repeatedly shown that consent documentation in surgical practice may be deficient in completeness and adherence to accepted standards. Deficiencies commonly reported include missing elements such as detailed descriptions of the procedure, personalised risks, alternatives, patient identifiers, and signatures of the patient and clinician. Such gaps in documentation can compromise patient understanding, weaken legal protection, and reflect systemic issues rather than isolated individual lapses [3].

Clinical audits of consent form completion have demonstrated that audit cycles with structured feedback and targeted interventions are effective in improving documentation quality. After implementing changes such as staff training, use of standardised consent checklists, or clarified documentation protocols, re-audit frequently reveals significant improvements in the presence of core consent elements and form completeness, highlighting quality improvement potential in consent processes [4].

In addition to formal audit results, observational studies and quality improvement reports support the need for regular evaluation and improvement of informed consent procedures. These studies emphasise that improving consent documentation not only enhances ethical and legal compliance but also strengthens patient safety practices and communication within clinical teams [5].

At Almanagil Teaching Hospital, Sudan, the completeness and quality of written surgical consent documentation had not previously been systematically evaluated. This quality improvement project was therefore undertaken to assess baseline compliance with accepted surgical consent standards, identify specific areas of poor documentation, implement targeted corrective interventions, and reassess performance through a second audit cycle, with the aim of improving patient safety, ethical practice, and documentation quality within surgical services.

## Materials and methods

Study design and setting

This project was conducted as a closed-loop quality improvement clinical audit designed to evaluate and improve the completeness of surgical consent documentation. The audit comprised a baseline assessment, implementation of a structured intervention, and a subsequent re-audit, allowing direct comparison of documentation practices before and after corrective measures. The study assessed routine clinical documentation without altering usual clinical workflows beyond the planned intervention. The audit was carried out at Almanagil Teaching Hospital, Sudan, a secondary-level teaching hospital providing both elective and emergency surgical services across multiple specialties.

Study period and population

The audit was conducted over a six-month period and included two audit cycles separated by an intervention phase. The baseline audit was performed between July and August 2025, during which 44 consecutive surgical consent forms completed using the existing consent format were reviewed. Following this, a two-month intervention phase was implemented between August and September. The re-audit was conducted between October and December 2025 and included 44 consecutive surgical consent forms completed after implementation of the intervention using the revised consent format. A total coverage sampling approach was used. All surgical consent forms completed during the specified audit periods were included, and the sample size (44 forms per cycle) reflected the available caseload and feasibility within the study timeframe rather than a formal power calculation.

Consent forms were eligible for inclusion if a written surgical consent document was present in the patient’s medical record and completed using the designated format relevant to the respective audit cycle. Forms that were missing, illegible, or completed using an inappropriate format were excluded to ensure consistency and validity of comparisons across audit cycles.

Audit standards and assessment criteria

Audit standards were informed by established ethical and legal principles of informed consent, institutional requirements, and best-practice guidance for surgical documentation. These standards guided the development of a structured audit checklist tailored to the documentation requirements and clinical workflow of Almanagil Teaching Hospital, while remaining aligned with internationally accepted consent principles. The checklist encompassed key domains of surgical consent documentation, including patient identification, surgical diagnosis and indication, procedural details, explanation of risks and alternatives, anaesthesia information, consent validity, professional accountability, and witness documentation where applicable. Each documentation element was assessed against predefined criteria, with a target compliance threshold of 80% applied to individual components, reflecting a pragmatic benchmark commonly adopted in quality improvement audits.

Data collection and quality assurance

Surgical consent forms were reviewed by trained doctors involved in institutional quality improvement activities. Prior to the data collection, reviewers underwent structured orientation and calibration to ensure consistent interpretation of audit criteria and uniform application of scoring rules. Each consent form was assessed using binary scoring, with documentation elements recorded as present or absent. Entries that were incomplete, unclear, or illegible were classified as absent to maintain strict scoring consistency.

Blinding of reviewers to the audit cycle was not feasible due to visible differences in the documentation formats before and after the intervention. To minimise observer bias, identical assessment criteria were applied across both cycles, and any discrepancies identified during review were resolved through consensus discussion. Formal inter-rater reliability testing was not undertaken, in keeping with pragmatic quality improvement methodology.

Intervention and re-audit

Following completion of the baseline audit, a structured intervention was implemented to address identified deficiencies in surgical consent documentation. This included targeted staff education on informed consent standards, feedback on baseline audit findings, and the introduction of a revised, structured surgical consent form designed to improve clarity and reduce omissions. Visual reminders and practical guidance were provided within clinical areas to support consistent completion of all consent components.

The re-audit reassessed surgical consent documentation using the same standards and criteria applied during the baseline cycle. This closed-loop approach enabled objective evaluation of the intervention’s impact, identifying areas of significant improvement as well as residual documentation gaps.

Data analysis

Data were collected using Google Forms (Google LLC, California, US) and analysed using both descriptive and inferential statistical methods. Documentation completeness for each consent component was summarised as absolute frequencies and percentages. Comparisons between cycle one and two were performed using the Chi-square test for categorical variables, with Fisher’s exact test applied where expected cell counts were less than five. Absolute percentage-point changes were calculated to quantify improvement between audit cycles. A p-value <0.05 was considered statistically significant. Analyses were conducted in keeping with the quality improvement nature of the project, and causal language was avoided.

Ethical considerations

This project involved review of routinely completed clinical documentation and was classified as a quality improvement audit rather than research involving human subjects. No direct patient contact occurred, and individual informed consent was not required. All data were anonymised prior to analysis, and no identifiable patient information was collected, stored, or reported. Ethical approval was obtained from the Institutional Review Board of Almanagil Teaching Hospital, Gezira State, Sudan (IRB Reference Number: IRB 25\MTH\1995). The project was conducted in accordance with institutional governance requirements and followed the Plan-Do-Study-Act methodology [6].

## Results

A total of 44 surgical consent forms were reviewed in each audit cycle. Baseline documentation completeness varied substantially across consent components, with particularly low compliance observed in patient identification fields, surgical indication, explanation of procedures and risks, consent validity indicators, and professional accountability elements. Following implementation of the structured quality improvement intervention, marked and statistically significant improvements were observed across nearly all domains of surgical consent documentation (Table 1).

**Table 1:** Comparative completeness of surgical consent documentation Audit done before and after a structured quality improvement intervention with chi-square analysis (cycle one vs cycle two, n=44 per cycle).

Documentation Item	Cycle 1 (n, %)	Cycle 2 (n, %)	Improvement (Δ%)	χ² value	p-value
Patient identification details					
Patient’s name	31 (70.5%)	42 (95.5%)	25	9.62	0.002
Hospital / File number	1 (2.3%)	42 (95.5%)	93.2	76.31	<0.001
Gender	1 (2.3%)	38 (86.4%)	84.1	69.18	<0.001
Diagnosis	12 (27.3%)	42 (95.5%)	68.2	48.77	<0.001
Surgical information					
Indication for surgery	2 (4.5%)	42 (95.5%)	91	74.05	<0.001
Procedure name	39 (88.6%)	42 (95.5%)	6.9	1.92	0.165
Site and side of surgery	14 (31.8%)	33 (75.0%)	43.2	16.21	<0.001
Explanation of procedure	7 (15.9%)	42 (95.5%)	79.6	63.94	<0.001
Possible additional procedures	3 (6.8%)	21 (47.7%)	40.9	17.86	<0.001
Anaesthesia and risk explanation					
Type of anaesthesia	31 (70.5%)	44 (100%)	29.5	15.37	<0.001
Anaesthesia risk explained	1 (2.3%)	43 (97.7%)	95.4	81.42	<0.001
Surgical risks explained	1 (2.3%)	41 (93.2%)	90.9	74.89	<0.001
Consent validity					
Consent documented	19 (43.2%)	44 (100%)	56.8	34.21	<0.001
Patient / representative name	44 (100%)	44 (100%)	0	—	—
Patient / representative signature	34 (77.3%)	44 (100%)	22.7	12.02	<0.001
Date documented	31 (70.5%)	44 (100%)	29.5	15.37	<0.001
Relationship of representative	15 (34.1%)	23 (52.3%)	18.2	3.05	0.081
Reason patient could not consent	2 (4.5%)	22 (50.0%)	45.5	20.74	<0.001
Physician details					
Physician name	16 (36.4%)	44 (100%)	63.6	41.98	<0.001
Physician position	1 (2.3%)	44 (100%)	97.7	84	<0.001
Physician signature	31 (70.5%)	44 (100%)	29.5	16.84	<0.001
Physician date & time	38 (86.4%)	44 (100%)	13.6	6.82	0.009
Witness details					
Witness name	32 (72.7%)	44 (100%)	27.3	14.12	<0.001
Witness signature	6 (13.6%)	44 (100%)	86.4	57.47	<0.001
Witness date & time	17 (38.6%)	44 (100%)	61.4	38.54	<0.001
Patient satisfaction confirmation					
Patient asked if satisfied	0 (0.0%)	44 (100%)	100	88	<0.001

Documentation of patient identification details improved significantly between audit cycles. Hospital/file number, gender, and diagnosis documentation increased from ≤26.8% at baseline to ≥86.4%-95.5% in cycle two, with all changes demonstrating statistical significance (p<0.001). Documentation of the patient’s name also showed a significant increase (71.4% to 95.5%, p=0.002).

Substantial improvements were observed in the surgical information domain. Documentation of the indication for surgery and explanation of the procedure increased from 5.4% and 16.1% at baseline to 95.5% post-intervention, respectively (p<0.001). Documentation of the surgical site and side improved significantly (32.1% to 75.0%, p<0.001). Although documentation of possible additional procedures remained comparatively lower (47.7% in cycle two), this still represented a statistically significant improvement relative to baseline (p<0.001). Documentation of the procedure name was high at baseline and did not demonstrate a statistically significant change (p=0.165).

In the anaesthesia and risk explanation domain, near-complete compliance was achieved following the intervention. Documentation of anaesthesia risks and surgical risks improved from 1.8% at baseline to over 93%-97% in cycle two, while documentation of anaesthesia type reached 100% compliance post-intervention (all p<0.001).

Indicators of consent validity demonstrated consistent enhancement. Confirmation that consent was documented increased from 42.9% to 100% (p<0.001), and completion of patient or representative signatures improved from 76.8% to full compliance (p<0.001). Documentation of the relationship of the representative showed modest improvement but did not reach statistical significance (p=0.081), whereas documentation of reasons for inability to consent improved significantly (5.4% to 50.0%, p<0.001).

Marked improvements were also evident in professional accountability and witness documentation. All physician identifiers, positions, signatures, and date/time fields reached 100% compliance in cycle two, with statistically significant improvements across all items (p<0.01). Witness documentation demonstrated similarly substantial gains, with witness signatures increasing from 14.3% to 100% (p<0.001).

Notably, patient satisfaction confirmation, which was entirely absent at baseline, achieved complete documentation (100%) following the intervention (p<0.001), representing the largest absolute improvement observed across all documentation domains.

## Discussion

This clinical audit demonstrated significant improvements across nearly all domains of surgical consent documentation at Almanagil Teaching Hospital following a structured quality improvement intervention. Baseline deficiencies were most pronounced in areas such as hospital/file number, gender, indication for surgery, risk explanation, consent validity elements, physician details, witness documentation, and patient satisfaction confirmation. Following targeted interventions, most consent components exhibited a marked increase in documentation completeness, with several domains achieving complete or near-complete compliance in the second audit cycle.

The findings of this audit align with international evidence showing that audit cycles with feedback and education can substantially enhance documentation quality. Leng et al. reported that re-auditing after targeted teaching sessions led to improved consent form completion, highlighting the effectiveness of structured audit and feedback in clinical practice improvement [1]. Similarly, Singh et al. identified baseline errors in consent documentation and demonstrated that targeted quality improvement measures significantly improved clarity and risk recording [7]. These studies support the assertion that audit-driven interventions are effective in addressing documentation gaps that may otherwise compromise informed consent quality.

The profound improvements observed in our audit, particularly for elements such as anaesthesia risk explanation and patient satisfaction confirmation, indicate that structured forms combined with education can transform routine clinical practice. This is consistent with literature emphasising the importance of well-structured consent tools. Altaf et al. found that comprehensive consent checklists improved documentation completeness in a surgical department, reinforcing the need for clear, standardised formats in busy clinical environments [5].

Despite the overall improvements, certain components remained suboptimal even after intervention, such as documentation of possible additional procedures and detailed reasoning when patients could not consent. This pattern resonates with findings from García-Álvarez et al., who observed persistent non-compliance in personalised risk information and complete physician identification in surgical consent forms, even in high-resource settings [8]. These areas may be inherently more challenging due to time constraints, clinician workload, or limited training focus, underscoring the need for ongoing reinforcement and perhaps supplementary decision aids or prompts within the consent process.

The ethical and legal imperatives for comprehensive informed consent cannot be overstated. Shafique et al. have underscored that informed consent is not merely a formality but a fundamental expression of patient autonomy and a safeguard against medico-legal repercussions when risks are inadequately conveyed or documented [2]. Moreover, incomplete consent documentation may contribute to broader communication gaps within the perioperative process, particularly in settings where multiple team members are involved in patient work-up and operative care, potentially increasing the risk of misunderstanding regarding the planned procedure and patient expectations [9].

Comparison with other settings illustrates variability in baseline practice and the universal need for structured improvement. Vieira et al. reported that less than half of the clinical files contained informed consent forms that met recommended guidelines, with many missing crucial details [10]. Similarly, Spatz et al. found significant variability in the quality of informed consent documentation across US hospitals, highlighting systemic challenges in meeting minimum documentation standards [11]. Our audit’s post-intervention results compare favourably with these findings and suggest that systematic audit cycles can bring practices closer to accepted standards even in resource-limited settings.

The observed improvements also have practical implications for patient care and safety. A more complete consent process promotes shared decision-making, clearer communication of risks and alternatives, and greater patient understanding and satisfaction. Patil et al. demonstrated that informed consent adequacy correlates with patient comprehension and satisfaction, which are critical to ethical care delivery [12].

Nonetheless, this audit has limitations that should be acknowledged. As with many clinical audits, the study design did not include a control group and was conducted at a single institution, which may limit generalisability. The reliance on documentation alone as a proxy for the consent process does not capture patient comprehension or the quality of verbal explanation, which future studies should address through qualitative methodologies or patient surveys. Readability and language comprehension of consent forms are also crucial factors, as highlighted by the systematic review by García-Álvarez et al., where poor readability was common and could impede truly informed consent [8].

In summary, this audit demonstrated that structured quality improvement interventions can substantially enhance the completeness of surgical consent documentation. Continued reinforcement, iterative audits, and complementary strategies such as training refreshers, multidisciplinary engagement, and usability improvements in consent forms may further consolidate gains and address persistent gaps.

## Conclusions

This closed-loop clinical audit demonstrated that a structured quality improvement intervention led to substantial improvements in the completeness of surgical consent documentation at the Almanagil Teaching Hospital. Most consent components showed marked improvement, with several achieving complete compliance following the intervention. The findings highlight the effectiveness of audit-driven interventions in strengthening adherence to informed consent standards, even in resource-limited settings. However, residual gaps in documenting possible additional procedures and details of patient incapacity indicate the need for continued reinforcement and further audit cycles. Routine auditing of surgical consent documentation should be embedded within the clinical governance processes to sustain improvements and ensure consistent, ethical, and high-quality surgical care.
